# The Analysis of Urban Nighttime Light Spatial Heterogeneity and Driving Factors Based on SDGSAT-1 Data

**DOI:** 10.3390/s26072094

**Published:** 2026-03-27

**Authors:** Jinke Liu, Yiran Zhang, Yifei Zhu, Xuesheng Zhao, Wei Guo

**Affiliations:** 1 College of Geoscience and Surveying Engineering, China University of Mining and Technology-Beijing, Beijing 100083, China; liujinkers@gmail.com (J.L.); zqt2300203108@student.cumtb.edu.cn (Y.Z.); zxs@cumtb.edu.cn (X.Z.); 2 State Key Laboratory for Tunnel Engineering, China University of Mining and Technology-Beijing, Beijing 100083, China; venuszyf0603@163.com

**Keywords:** SDGSAT-1, geographically weighted random forest, SHAP, nighttime light

## Abstract

**Highlights:**

1. Application of SDGSAT-1 high-resolution nighttime light data to analyze street-scale ALAN spatial heterogeneity in Beijing and Guangzhou, revealing city-specific lighting patterns.
2. Novel integration of Geographically Weighted Random Forest (GWRF) and SHAP to capture local variations in ALAN intensity, identifying non-linear relationships and providing deeper insights into urban lighting drivers.

**What are the main findings?**

- Road density is the dominant factor influencing nighttime light intensity in both Beijing and Guangzhou. However, the relationship between ALAN and its driving factors varies across these cities.
- In Beijing, a balanced multi-factor model is observed, whereas in Guangzhou, ALAN intensity is primarily driven by road density, with secondary influences from other factors like sky view factor.

**What are the implications of the main findings?**

- The integration of high-resolution SDGSAT-1 data with GWRF and SHAP offers a powerful framework for spatially adaptive urban analysis, enabling more precise monitoring of urban development, socio-economic activities, and light environment management.

**Abstract:**

Artificial light at night (ALAN) data is widely used in urban function analysis and socio-economic activity monitoring, but its application at the micro-scale of cities still faces challenges. This study utilizes high spatial resolution SDGSAT-1 nighttime light data to explore the spatial heterogeneity of ALAN at the street scale in two representative Chinese cities—Beijing and Guangzhou. By integrating multi-source data (such as building vector data, road networks, and point of interest data), a multi-dimensional indicator system covering urban morphology, functional structure, and transportation accessibility is constructed. Based on this, the study employs a Geographically Weighted Random Forest (GWRF) model combined with the Shapley Additive Explanations (SHAP) method to deeply analyze the non-linear relationships between ALAN intensity and multiple driving factors, as well as their spatial variability. Results demonstrate the superiority of the GWRF model over global models in capturing spatial non-stationarity, with R^2^ values of 0.67 for Beijing and 0.74 for Guangzhou, compared to 0.62 and 0.71 for the random forest models, respectively. Road density is the dominant factor influencing nighttime light intensity in both Beijing and Guangzhou. However, the relationship between ALAN and its driving factors varies across these cities. In Beijing, a balanced multi-factor model is observed, whereas in Guangzhou, ALAN intensity is primarily driven by road density, with secondary influences from other factors like sky view factor. This study validates SDGSAT-1 for micro-scale analysis, offering a scientific basis for differentiated urban lighting planning.

## 1. Introduction

With the rapid advancement of global urbanization, artificial light at night (ALAN) has become an important proxy for human activities, urban structure, and socioeconomic dynamics [1,2,3,4]. Nighttime light data have been widely used to characterize urban expansion, economic activity, energy consumption, and disaster recovery due to their broad spatial coverage and temporal continuity [5,6,7,8,9,10,11]. However, most existing studies rely on moderate- to coarse-resolution datasets (e.g., DMSP/OLS and VIIRS), which are more suitable for macro-scale analysis and often fail to capture fine-scale spatial heterogeneity within cities [12,13,14,15,16].

This limitation becomes particularly critical at the intra-urban scale, where lighting patterns are strongly shaped by street morphology, land use, and transportation infrastructure. Low-resolution data are prone to mixed pixels, blooming effects, and loss of spatial detail [17,18,19], making them insufficient for analyzing micro-scale urban processes such as street-level activity intensity, lighting infrastructure distribution, and local environmental conditions [20,21,22]. As a result, understanding the fine-grained spatial mechanisms of ALAN remains a key challenge.

In recent years, the emergence of a new generation of high-resolution ALAN datasets has created significant opportunities for research at the urban micro-scale [23,24]. For example, the JL1-3B satellite can provide meter-level RGB nighttime imagery, enabling extremely detailed observation of urban lighting structures [25,26]. However, such commercial data are often constrained by acquisition costs and task-based imaging schedules, which may limit their use in large-scale or reproducible studies. In contrast, SDGSAT-1 provides 10–40 m multispectral nighttime observation data through an open-sharing policy [27,28,29,30,31]. Being free and easily accessible, it is better suited to support reproducible research on urban fine-grained ALAN mechanisms and frameworks for routine monitoring [32,33,34].

At the same time, the formation of ALAN is inherently complex. Beyond macro-level factors such as population and economic activity, it is closely associated with urban morphology, functional structure, and transportation accessibility [35,36,37,38]. These relationships are often nonlinear and spatially heterogeneous, meaning that the influence of the same factor may vary significantly across locations [39]. Traditional global models and standard machine learning approaches typically assume spatial stationarity and therefore struggle to capture such local variations [40,41,42,43]. To address these challenges, this study integrates a geographically weighted random forest (GWRF) model with SHAP (Shapley Additive Explanations). The GWRF model enables the capture of spatial non-stationarity by incorporating local spatial weighting into a nonlinear machine learning framework [44,45,46,47], while SHAP provides interpretable insights into the contribution and interaction of driving factors at different locations [48,49,50].

Based on this, this study selects Beijing and Guangzhou as representative megacities for empirical analysis. Using SDGSAT-1 high-resolution nighttime light imagery to characterize street-scale ALAN intensity, we integrate multi-source spatial data to construct a multidimensional indicator system representing urban morphology, functional structure, and transportation accessibility. Building upon this framework, a geographically weighted random forest (GWRF) model is applied to capture the complex nonlinear relationships and spatial non-stationarity between ALAN intensity and these driving factors. Furthermore, SHAP is introduced to interpret the model results by revealing the local contributions, threshold effects, and interactions of key variables under different urban contexts, thereby providing a spatially explicit and interpretable framework for understanding the formation mechanisms of urban nighttime activities.

## 2. Materials and Methods

### 2.1. Study Area

**Figure 1:**
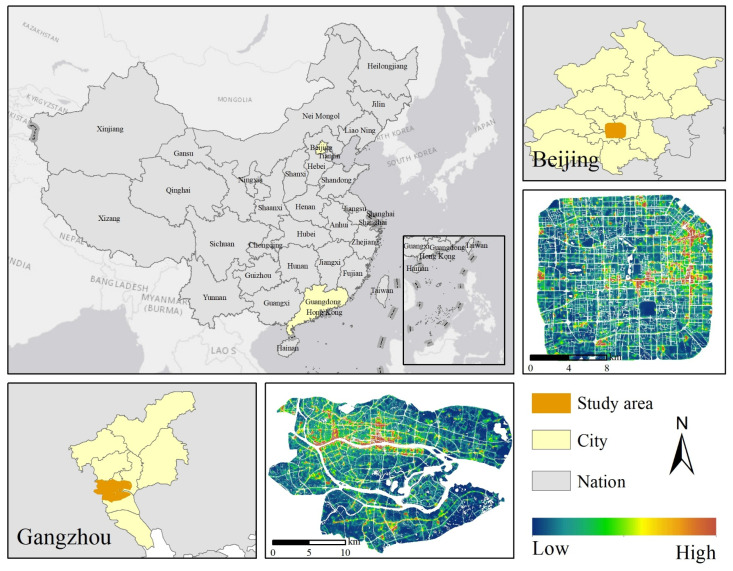
Geographical location of the study areas and SDGSAT-1 nighttime light map: Beijing and Guangzhou.

This study selects Beijing and Guangzhou as representative case studies to investigate the formation mechanisms of urban ALAN spatial heterogeneity at the street scale (Figure 1). Although both cities serve as core economic growth poles in China, they exhibit significant differences in development stages, spatial morphological evolution, and the characteristics of nocturnal socio-economic activities. These distinctions provide a highly representative comparative perspective for deeply analyzing the driving factors and spatial response patterns of ALAN distribution under different urban contexts.

Beijing (39°28′ N–41°05′ N, 115°22′ E–117°30′ E), located at the northwestern edge of the North China Plain, serves as the national center for politics, culture, and technological innovation. In recent years, driven by strategies such as the relief of non-Capital functions and urban renewal, Beijing’s spatial morphology is evolving from a monocentric structure to a multi-node agglomeration pattern. This unique political and spatial background profoundly influences its ALAN landscape.

Guangzhou (22°26′ N–23°56′ N, 112°57′ E–114°03′ E) is situated in the core of the Pearl River Delta, serving as a national central city and the economic engine of the Greater Bay Area. As a renowned commercial hub in South China, Guangzhou is famous for its vibrant nighttime economy. Its night market culture, large-scale commercial complexes, and nocturnal traffic flows jointly constitute a rich and diverse ALAN landscape.

### 2.2. Data Collection

To elucidate the spatial heterogeneity of SDGSAT-1 nighttime light, this study integrates multi-source spatial data to construct a street-scale urban spatial indicator system. The primary datasets employed include Point of Interest (POI) data, SDGSAT-1 ALAN remote sensing imagery, OpenStreetMap (OSM) road network data, and building vector data. Detailed descriptions of each dataset are as follows:

The 2024 SDGSAT-1 satellite nighttime light data were obtained from the International Research Center of Big Data for Sustainable Development Goals (https://www.sdgsat.ac.cn/, accessed on 21 May 2025). The dataset comprises one panchromatic band (PL/PH) and three multispectral bands (R, G, B), with spatial resolutions of 10 m and 40 m, respectively. We utilized the Level-4 (L4) products, which have undergone rigorous preprocessing including geometric correction, radiometric calibration, and orthorectification. The RGB bands were preferred over the panchromatic band due to their superior geometric stability and suitability for quantitative analysis. This study selects the RGB multispectral bands as the primary data source.

The 2024 POI data were acquired via the AutoNavi Map (Amap) open platform (https://www.amap.com/, accessed on 21 May 2025). POI data effectively characterize the spatial distribution of human activities and urban functional attributes, serving as a vital fine-grained data source for identifying urban functional structures. This study utilizes POI data covering diverse categories—including dining, retail, entertainment, education, medical services, and office spaces—and extracts key attributes such as names, categories, geographic coordinates, and classification levels. Due to their high update frequency and extensive spatial coverage, these data realistically reflect the intensity of urban activities, functional organization, and consumer behavior characteristics. Consequently, POI data are employed as a significant driving factor to explain the spatial differentiation of ALAN intensity.

Building footprint vector data of the year 2024 are used to characterize the morphological structure of the built environment and provide a fundamental basis for understanding the spatial logic of urban nighttime light emissions. In this study, we employed building vector data obtained from the AutoNavi Map (Amap) Open Platform (https://www.amap.com/, accessed on 27 May 2025), which include building outlines, footprint area, and floor/height information. These data enable a street-level characterization of building density, height distribution, and urban form.

The road network data of the year 2024 were derived from OpenStreetMap (OSM) (https://www.openstreetmap.org/, accessed on 29 May 2025), an open geographic information platform collaboratively maintained by global users, characterized by high accessibility, timely updates, and fine spatial granularity. This dataset includes detailed attributes such as road types, hierarchy levels, node positions, and traffic-related properties, comprehensively representing the urban road morphological structure and traffic connectivity.

The street data used in this study are derived from the EULUC-China 2.0 (Essential Urban Land Use Categories in China) dataset, which has been widely applied in urban land-use studies across multiple cities in China [51]. The EULUC-China dataset is developed in accordance with the Classification of Land Use Status (GBT 21010-2017) standard and provides parcel-level functional land-use information for major Chinese cities [52]. It should be noted that, in this study, the street data do not incorporate explicit functional attributes. Instead, street units are constructed based on vector street data obtained from OpenStreetMap.

## 3. Methods

**Figure 2:**
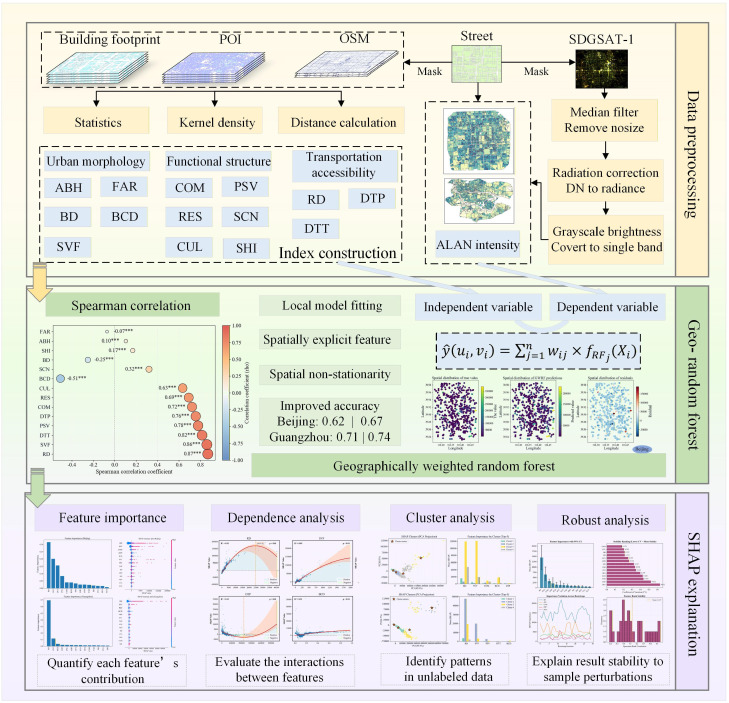
The framework for investigating SDGSAT-1 nighttime light heterogeneity based on the GWRF and SHAP (*** indicates significance at the 0.001 level).

This study proposes a street-level ALAN analysis framework that integrates SDGSAT-1 high-resolution nighttime light imagery with geographically weighted random forest modeling and SHAP-based interpretation. First, we develop a multidimensional indicator system encompassing urban morphology, functional structure, and transportation-related factors to comprehensively characterize urban spatial structure. We then employ the GWRF model to address spatial non-stationarity in the relationships between ALAN and its potential drivers. Finally, SHAP is used to enhance model interpretability by quantifying the local contributions of individual factors across streets, thereby revealing nonlinear responses and potential threshold effects among variables (Figure 2).

### 3.1. SDGSAT-1 Nighttime Light Data Preprocessing

SDGSAT-1 observations can be affected by sensor noise, cosmic-ray hits, atmospheric scattering, and spatially varying surface reflectance, which may generate band-inconsistent outliers. If left uncorrected, these anomalous pixels can compromise radiometric consistency and propagate errors into subsequent modeling. To mitigate noise effects from sensor artifacts and cosmic-ray hits, a median filter with a 5 × 5 pixel window was applied to denoise the SDGSAT-1 nighttime light imagery. This nonlinear filter replaces each pixel with the median value within its neighborhood, effectively suppressing isolated spikes while preserving brightness gradients and structural details such as urban edges and road corridors.

Following the denoising step, we carried out band-wise radiometric calibration for the three RGB channels to convert digital numbers (DN) into physically meaningful radiance. DN represents the original digital count recorded by the satellite sensor; gain is the radiometric conversion coefficient that scales DN values to physical radiance units; bias is the offset term used to correct for sensor baseline signal; wavelength indicates the spectral response range of each band. Using the sensor-provided gain and bias coefficients, DN values were transformed to radiance (W/m^2^/sr/μm) according to the SDGSAT-1 calibration relationship, expressed as follows:

(1) $${Lightness}_{SDGSAT-1}=DN\times Gain+Bias$$

Among them, ${Lightness}_{SDGSAT-1}$ represents the radiometric brightness value of the SDGSAT-1 nighttime light image, with the unit being W/m^2^/sr/μm. The relevant parameter table is shown as follows (Table 1):

To further quantify the integrated radiative intensity across the RGB channels, we fused the band-specific radiance values to derive a single grayscale luminance metric that represents the overall optical energy level of SDGSAT-1 nighttime observations. In this study, we adopted the color-to-grayscale conversion method proposed by Grundland [53]. The grayscale luminance (W/m^2^/sr/μm) of SDGSAT-1 nighttime imagery is computed as follows:

(2) $${Brightness}_{SDGSAT-1}=0.2989\times C_{R}+0.5870\times C_{G}+0.1140\times C_{B}$$

where $C_{R}$, $C_{G}$, and $C_{B}$ denote the radiance values of the red, green, and blue bands of the SDGSAT-1 nighttime light data, respectively.

### 3.2. Index Construction

To reveal the spatial heterogeneity of urban ALAN distribution and its driving mechanisms, this study constructs a multidimensional indicator system that comprehensively characterizes key factors influencing ALAN intensity from three dimensions: urban morphology, functional structure, and transportation accessibility (Table 2). The construction and significance of each group of indicators are described below.

The urban morphology indicators include floor area ratio, average building height, building density, building concentration density, and sky view factor. These metrics are used to quantify building density, vertical development, and the geometric configuration of the built environment. The formulas and descriptions of the indicators are presented in Table 3.

The functional structure indicators consist of commercial density, public service density, residential density, scenery density, cultural density, and the Shannon index (Table 4). These indicators are designed to capture the intensity and diversity of human activities and functional composition, which are closely associated with nighttime lighting demand. Kernel density estimation (KDE) characterizes the spatial aggregation of analysis targets and reflects clustering intensity, thereby providing valuable spatial insights and decision support for analyzing urban patterns. A higher kernel density value indicates a denser concentration of POIs in a given area. In this study, KDE was applied to qualitatively analyze five categories of POI elements, visually mapping the spatial hotspots of each category to illustrate their distributional characteristics. To capture the functional complexity of urban areas, we calculated the Shannon diversity index (SHDI) based on the distribution of POI categories.

The transportation accessibility indicators include road density, distance to parking facilities, and distance to transport stations. These variables characterize the density of the urban road network and the average proximity of streets to parking facilities and public transport nodes (e.g., metro and bus stations), reflecting the influence of transportation infrastructure and mobility on nighttime light intensity.

### 3.3. Spearman Correlation Coefficient

Spearman correlation is a non-parametric statistical method. Its core mechanism involves converting two sets of raw data into ranks (i.e., the ordinal position of data after sorting, where tied values are assigned the average rank) and subsequently calculating the correlation based on these ranks. Unlike parametric methods, it does not rely on the assumption of a normal distribution, making it particularly suitable for analyzing non-normally distributed continuous data or ordinal data. Compared to Pearson correlation, which depends on specific distributional assumptions, Spearman correlation is more robust to outliers and applicable to a broader range of scenarios. The calculation formula is expressed as follows:

(3) $$P=1-\frac{6\sum_{i=1}^{n}(x_{i}-y_{i}{)}^{2}}{n\left(n^{2}-1\right)}$$

where P denotes the spearman correlation coefficient, with a value range of [−1, 1]. Specifically, P = 1 indicates a perfect positive correlation, P = −1 signifies a perfect negative correlation, and P = 0 implies no monotonic correlation between the two datasets. $x_{i}$ and $y_{i}$ represent the ranks of the ith observation for variables x and y, respectively, and n denotes the sample size.

### 3.4. Geographically Weighted Random Forest

Geographically weighted random forest (GWRF) represents a hybrid spatial analysis methodology that integrates the non-linear modeling robustness of the random forest algorithm with the local parameter estimation philosophy of GWR. Primarily designed to address spatial heterogeneity, the underlying mechanism of GWRF relies first on the random forest architecture. Random forest is an ensemble learning technique that performs regression or classification by constructing a multitude of decision trees and aggregating their predictions. Through bootstrap sampling (sampling with replacement) and random feature selection at each split node, this algorithm effectively mitigates overfitting, enhances generalization stability, and captures complex non-linear interactions among high-dimensional features. To extend this framework into the spatial domain, GWRF incorporates the core tenet of GWR: the recognition of spatial non-stationarity, where relationships between variables drift across geographic locations. Unlike global models that assume a constant relationship, GWRF assigns distinct spatial weights to observations based on their proximity to a target estimation point. Following the principle of distance decay, samples closer to the target location are assigned higher weights during the training process, while distant samples exert minimal influence.

Operationally, the algorithm constructs a separate, locally weighted random forest model for every specific location (or sampling point) within the study area. By prioritizing neighboring samples through this distance-based weighting scheme, each local model is tailored to fit the specific data characteristics of its immediate vicinity. This architecture allows GWRF to effectively decompose complex spatial structures and capture local variations that global models often overlook, making it a powerful analytical tool for fields such as geography, environmental science, and ecology [54,55,56]. The fundamental formulation of GWRF is expressed as follows:

(4) $$\hat{y}\left(u_{i},v_{i}\right)=\sum_{j=1}^{n}w_{ij}\times f_{{RF}_{j}}(X_{i})$$

where $\left(u_{i},v_{i}\right)$ denotes the geographic coordinates of the ith sample. $w_{ij}$ represents the spatial weight of the sample relative to the prediction point. $f_{{RF}_{j}}$ signifies the local random forest model trained in the vicinity of the specific location. $X_{i}$ is the feature vector of the ith sample.

For each target street unit, Euclidean distances to all samples were calculated and spatial weights were assigned using a Gaussian kernel function. An adaptive bandwidth strategy was adopted, where the bandwidth was determined by the 300 nearest neighboring samples used to construct each local model. The local prediction model was implemented using a Random Forest Regressor, with the number of trees (n_estimators) set to 300, the maximum tree depth (max_depth) set to 100, and the minimum number of samples per leaf (min_samples_leaf) set to 1. Gaussian kernel weights were incorporated as sample weights during model training. To ensure reproducibility, a fixed random seed of 44 was used in both model training and dataset shuffling.

To further evaluate the robustness and generalization performance of the model, a five-fold cross-validation strategy was employed. Specifically, the entire dataset was randomly partitioned into five equal subsets, of which four subsets were used for training and the remaining one for validation in each iteration. This process was repeated five times so that each subset was used exactly once as the validation set. Model performance was assessed using metrics including the coefficient of determination (R^2^) and root mean square error (RMSE). The final evaluation results were obtained by averaging the performance metrics across all five folds, providing a reliable estimate of model predictive accuracy and stability.

### 3.5. SHAP

SHAP (Shapley Additive Explanations) is an interpretability method designed to explain the predictions of machine learning models. Its core principle is grounded in the Shapley value concept from cooperative game theory. In the context of a cooperative game, the Shapley value measures the average marginal contribution of each player to the total payoff. When adapted to machine learning, the model’s prediction is analogous to the total payoff of the game, while the input features are treated as the participating players. Consequently, the Shapley value quantifies the average marginal contribution of a specific feature to the final prediction value across all possible feature combinations (or coalitions).

The Shapley value of the ith feature is defined as:

(5) $$\phi =\sum_{S\subseteq N\backslash \{i\}}\frac{\left|S\right|!\left(\left|N\right|-\left|S\right|-1\right)!}{\left|N\right|!}[f_{x}\left(S\cup \left\{i\right\}\right)-f_{x}(S)]$$

where N is the set of all features; S represents a subset of features that excludes the ith feature; $f_{x}(S)$ denotes the prediction output using only the feature set S; and the term serves as a weighting coefficient, representing the probability of the subset S occurring.

(6) $$f\left(x\right)\approx g\left(x^{\prime }\right)=\phi_{0}+\sum_{i=1}^{M}\phi x_{i}^{\prime }$$

where $f\left(x\right)$ represents the prediction of the original complex model for the instance; $g\left(x^{\prime }\right)$ denotes the explanation model; $x^{\prime }$ is the simplified input representing the presence or absence of a feature; M is the number of simplified input features; and $\phi_{0}$ is the baseline value.

## 4. Results

### 4.1. The Correlation Between ALAN and Explanatory Variables

**Figure 3:**
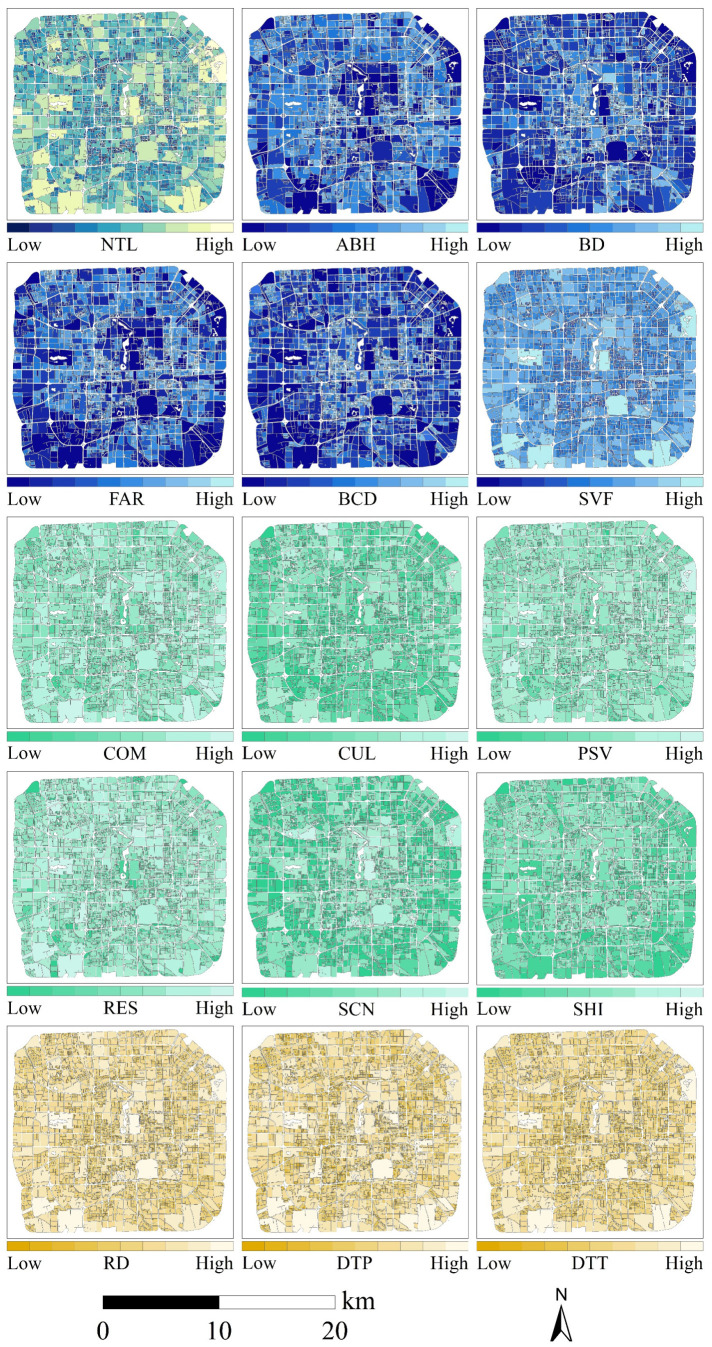
Spatial distribution map of the street-scale ALAN and its driving factors in Beijing.

**Figure 4:**
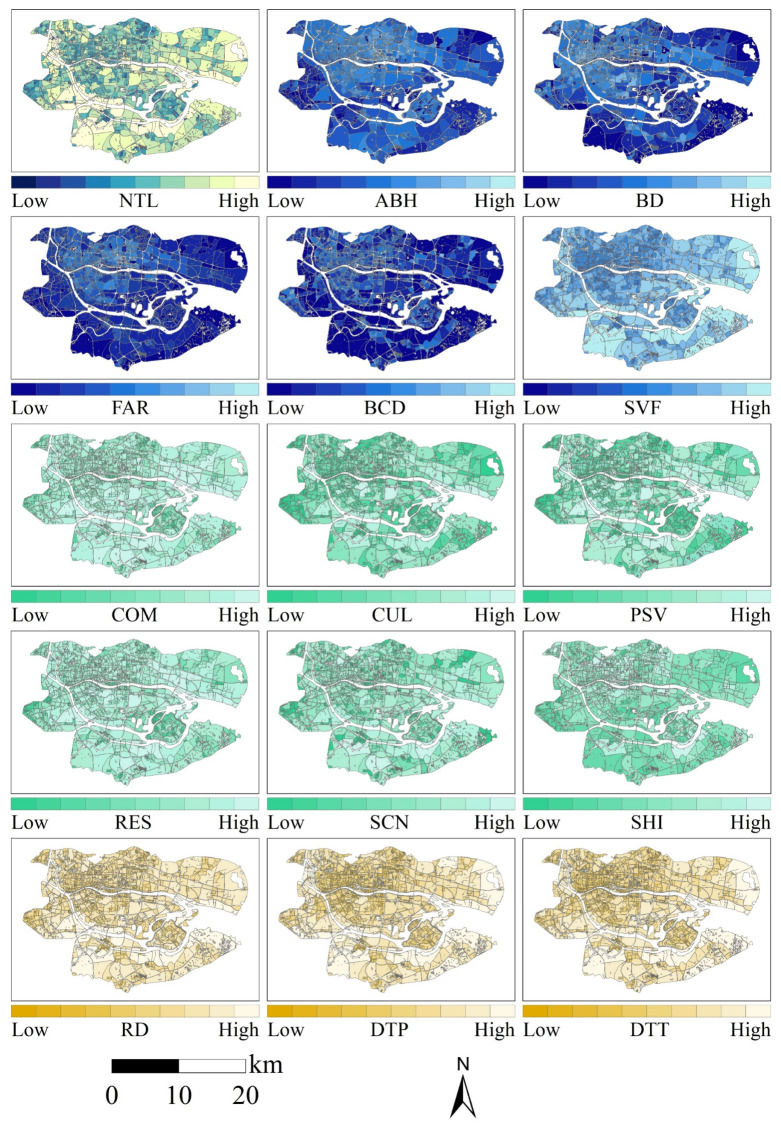
Spatial distribution map of the street-scale ALAN and its driving factors in Guangzhou.

**Figure 5:**
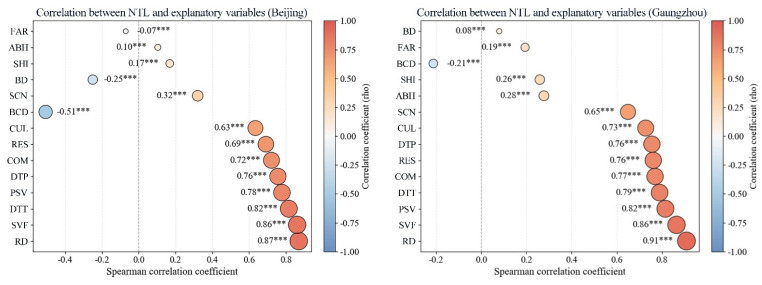
Correlation between ALAN intensity and explanatory variables (*** indicates significance at the 0.001 level).

To preliminarily identify the primary determinants of urban street nighttime light intensity, this study utilized SDGSAT-1 high-resolution ALAN imagery and employed the Spearman correlation coefficient to conduct a statistical correlation analysis. The analysis examined the relationship between total ALAN within city blocks of the two cities and a multi-dimensional set of indicators covering urban morphology, functional structure, and transportation accessibility (Figure 3 and Figure 4). Overall, ALAN in both cities exhibited significant positive correlations with road density (RD), sky view factor (SVF), public service density (PSV), distance to public transport (DTT), distance to parking facilities (DTP), and commercial density (COM), with correlation coefficients exceeding 0.70 (*p* < 0.001) (Figure 5).

### 4.2. Fitting Performance of the Geographically Weighted Random Forest (GWRF) Model

**Figure 6:**
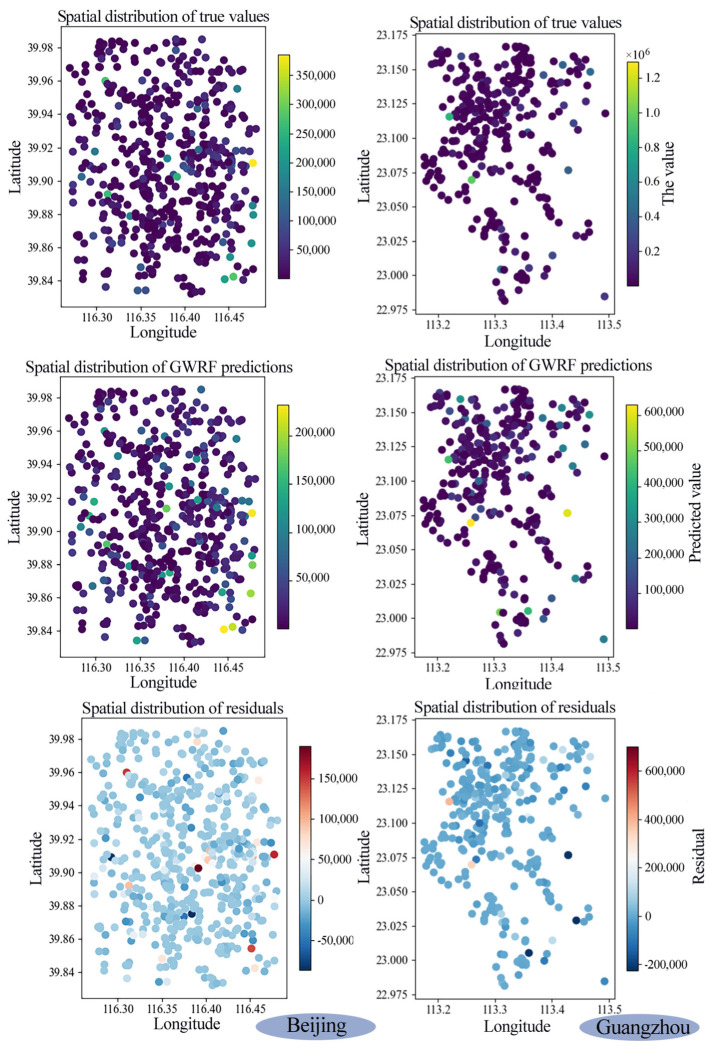
The spatial distribution of the true values, the predicted values of GWRF, and the residuals are shown in Beijing and Guangzhou.

Figure 6 presents a comprehensive overview of the fitting performance and spatial characteristics of the GWRF model for street-level ALAN regression in Beijing and Guangzhou. Compared with the global random forest model, the GWRF model achieves slightly higher R^2^ values (0.67 in Beijing and 0.74 in Guangzhou for GWRF versus 0.62 and 0.71 for RF) and lower RMSE values (27,152 in Beijing and 45,644 in Guangzhou for GWRF versus 28,926 and 47,223 for RF). The geographically weighted framework enables the model to capture spatial non-stationarity in the relationships between ALAN intensity and urban environmental factors, providing a foundation for spatially explicit interpretation of the underlying driving mechanisms. An analysis of the residuals reveals that discrepancies between observed and predicted values are minimal for the majority of samples, with outliers confined to localized anomalies rather than systematic spatial clusters. The Moran’s I value of the residuals in Beijing is 0.029 (*p* = 0.26 > 0.05), while in Guangzhou it is −0.002 (*p* = 0.46 > 0.05). For both Guangzhou and Beijing, the residuals do not exhibit significant spatial autocorrelation. These findings indicate that GWRF successfully captures the local interplay between ALAN and urban morphology, function, and traffic metrics. While slight deviations persist in areas with distinct lighting patterns or rapid development, the model demonstrates overall robustness, establishing a solid foundation for the subsequent SHAP-based interpretation of driving mechanisms.

### 4.3. Feature Importance Analysis and SHAP Interpretation

**Figure 7:**
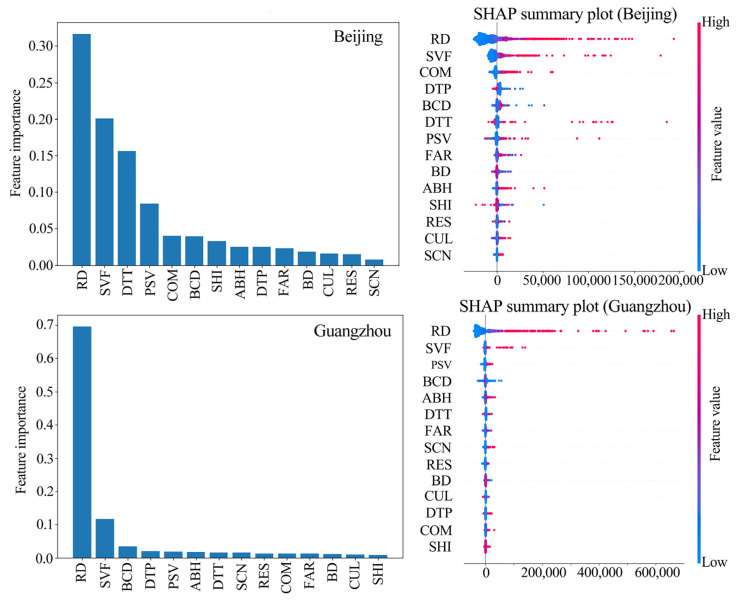
Feature importance and SHAP summary plot for Beijing and Guangzhou.

A combined analysis of feature importance and SHAP summary plots reveals that while Beijing and Guangzhou share primary determinants for street-scale ALAN, their secondary driving architectures diverge significantly (Figure 7). The feature importance rankings (left column) categorize Guangzhou as a traffic-dominated model, where RD commands an overwhelming importance weight of ~0.70. In contrast, Beijing exhibits a multi-factor model, characterized by a more balanced distribution among RD (~0.32), SVF (~0.20), and DTT (~0.15).

RD ranks as the most critical variable in both cities. The SHAP point clouds are predominantly distributed in the positive range, where high feature values correspond to large positive SHAP values. This indicates that streets with denser networks and higher hierarchy levels exhibit significantly elevated ALAN intensity. Samples with high SVF values (red dots, representing high spatial openness) are predominantly concentrated in the positive SHAP value range, whereas those with low values (blue dots, representing severe building occlusion) are clustered in the negative range. High SVF typically corresponds to high-grade arterial roads, broad intersections or open commercial plazas, which are generally equipped with high-intensity municipal lighting. In contrast, low SVF is often found in narrow alleys or aging neighborhoods characterized by extremely high building density. Constrained by the lower road hierarchy, public lighting facilities in these areas are relatively scarce, resulting in lower ALAN intensity.

**Figure 8:**
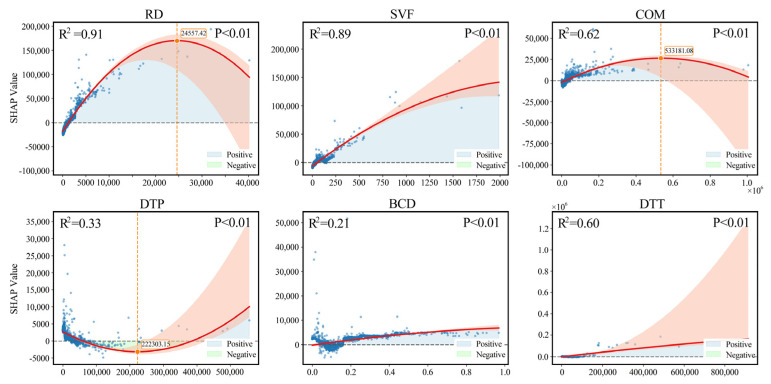
SHAP dependence plots of the primary driving factors for night-time light in Beijing. The orange part represents the 95% confidence interval.

Figure 8 illustrates the SHAP dependence plots for the primary driving factors in Beijing. RD emerges as the most significant explanatory variable for ALAN variation, exhibiting an inverted U-shaped response. In low-to-medium density zones, SHAP values rise rapidly, reflecting the combined boost from road lighting, commercial vitality, and traffic flow. However, beyond a threshold of approximately 2.4 × 10^4^, SHAP values decline, indicating that lighting within ultra-dense road networks approaches saturation, thereby diminishing the marginal contribution of additional roads to brightness. The SVF shows a near-linear positive correlation. This suggests that greater spatial openness enables fuller horizontal light diffusion and facade reflection, acting as a critical structural amplifier for Beijing’s ALAN. COM also displays a typical inverted U-shaped curve. Although BCD makes a smaller contribution, it maintains a gradual upward trend. Conversely, DTT exhibits a significant exponential increase. While ALAN remains stable near transit stations, SHAP values surge in areas distant from the rail system. This underscores that traffic corridors and vehicular mobility are dominant drivers of luminosity in Beijing’s periphery.

**Figure 9:**
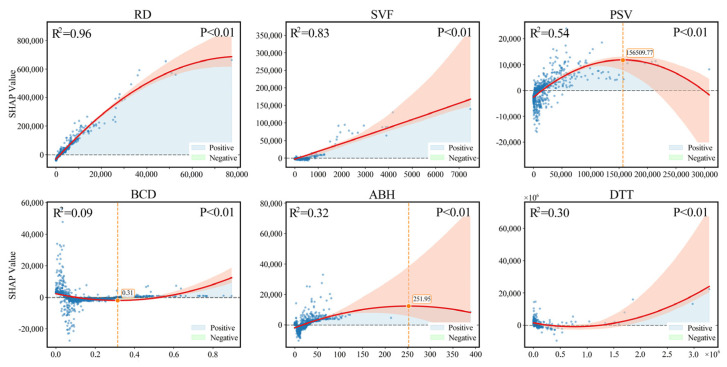
SHAP dependence plots of the primary driving factors for night-time light in Guangzhou. The orange part represents the 95% confidence interval.

Figure 9 illustrates the SHAP dependence plots for the primary driving factors of ALAN intensity in Guangzhou. The overall response characteristics reveal the core mechanisms of urban ALAN formation. In contrast to the balanced multi-factor structure observed in Beijing, Guangzhou is characterized by a dominance of RD and SVF, with public services and building height playing secondary roles.

RD emerges as the most dominant explanatory variable, exhibiting an approximately monotonic upward trend (R^2^ = 0.96). In low-density networks, increased RD significantly elevates SHAP values, suggesting that street lighting, commercial facades, and traffic flow jointly establish the baseline brightness. Although the growth rate diminishes in high-density zones, the continued rise reflects the structural dependence of Guangzhou’s ALAN on traffic corridors. Similarly, the SVF demonstrates strong explanatory power (R^2^ = 0.84) with a linear growth pattern. This indicates that higher spatial openness facilitates wider light observation, a positive enhancement that is particularly pronounced given Guangzhou’s abundant waterfronts and wide road profiles.

Secondary factors such as PSV and ABH display inverted U-shaped relationships (R^2^ = 0.53 and R^2^ = 0.32, respectively). Moderate agglomeration of services and building height significantly enhances brightness; however, marginal contributions plateau or decline beyond specific thresholds. This trend suggests that in ultra-dense areas, building layouts and potential light scattering effects may limit further ALAN gains. Finally, DTT presents a distinct U-shaped structure (R^2^ = 0.30). Areas in close proximity to rail stations exhibit weak ALAN contributions, reflecting their primary function as commuting hubs. Conversely, SHAP values surge at distances beyond 800 m, indicating that ALAN in peripheral zones is driven primarily by vehicular traffic and expressways rather than public transit nodes.

### 4.4. Clustering of ALAN Driving Mechanisms and Robustness Analysis

**Figure 10:**
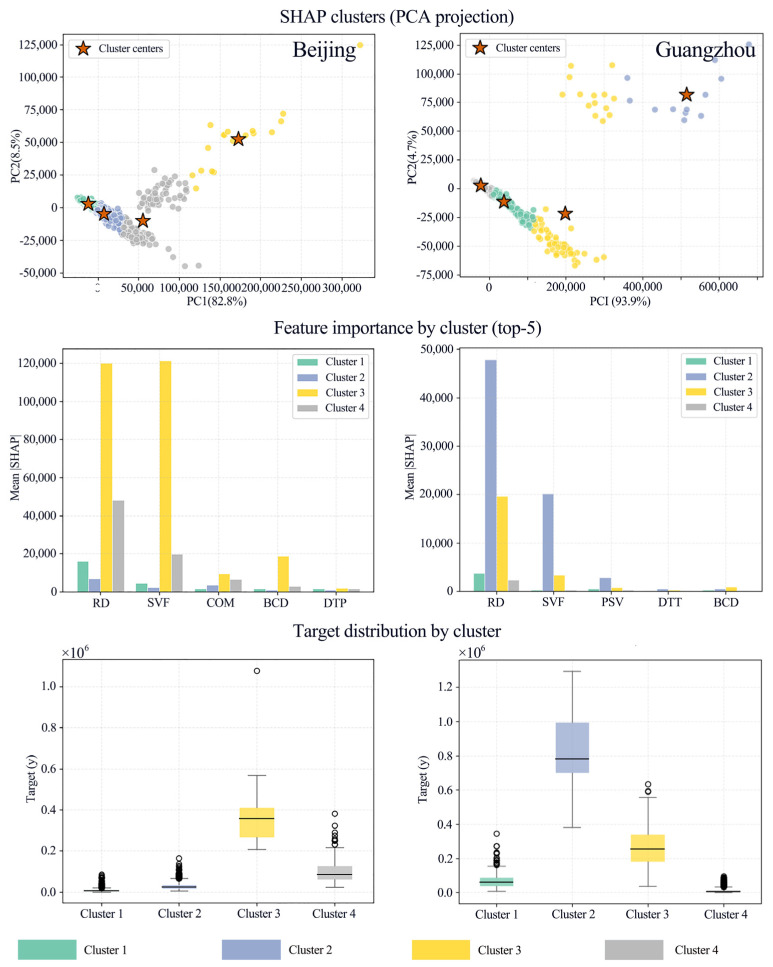
Comparative analysis of spatial heterogeneity in nighttime light driving mechanisms between Beijing and Guangzhou based on SHAP clustering.

To further operationalize the spatial non-stationarity of ALAN driving mechanisms and to empirically distinguish the city-specific ALAN formation patterns in Beijing and Guangzhou, we conducted a cluster analysis based on SHAP values of all driving factors. This analysis partitions street units into four distinct clusters, each representing a unique combination of factor contributions to ALAN intensity, and the spatial agglomeration of clusters directly reflects the heterogeneous distribution of ALAN driving mechanisms across urban spaces. Figure 10 presents the clustering results based on SHAP values, revealing a significant heterogeneous structure of samples within the model explanation space. For Beijing, PCA projections indicate that the primary differences in SHAP representations unfold along the first principal component (PC1), which explains 82.8% of the variance (compared to only 8.5% for PC2). The clear separation of clusters within the PCA space suggests distinct dominant gradients in the explanatory mechanisms of the samples. Regarding the distribution of the target variable (y-value), the clusters exhibit distinct hierarchical characteristics. Cluster 1 (60.3%) and Cluster 2 (31.8%) constitute the baseline population, with target values concentrated at low-to-medium levels. Cluster 4 (7.2%) represents a transitional state with medium-to-high values. Although Cluster 3 (0.7%) accounts for a very small proportion, its median and interquartile range are significantly elevated, accompanied by extreme outliers, clearly corresponding to the high-value or extreme states of the target variable. In Guangzhou, the clustering results show stronger linear separation along the principal PCA axis, with PC1 explaining 93.9% of the variance, indicating that its explanatory structure converges more notably upon a single dominant gradient. Inter-cluster stratification in Guangzhou is also more rigorous: Cluster 2 corresponds to the highest target value level, followed by Cluster 3, then Cluster 1, with Cluster 4 being the lowest. This reflects a high degree of concentration in the explanatory mechanisms for high-value samples.

**Figure 11:**
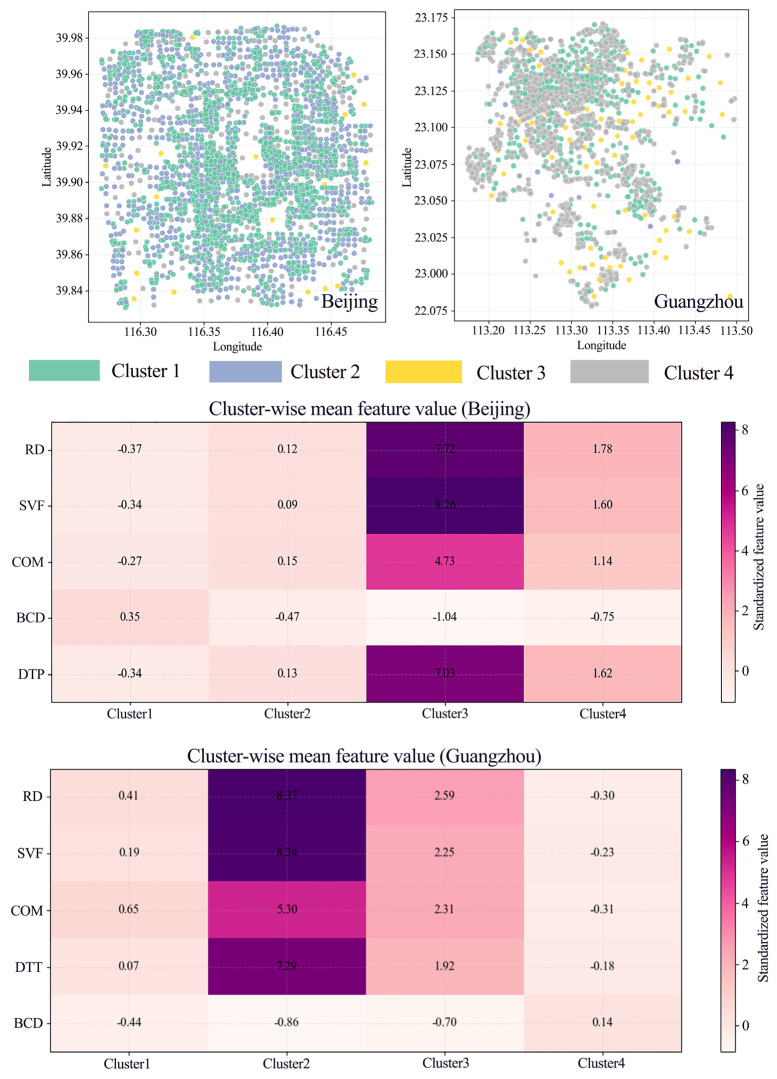
The spatial scatter plots and cluster-wise mean feature values for Beijing and Guangzhou.

The analysis of intra-cluster feature contributions (Mean |SHAP|) further reveals differences in dominant drivers across sample levels. In Beijing, the high-value cluster (Cluster 3) demonstrates a strong dependence on a few key factors for model prediction, as its mean |SHAP| magnitude for RD and SVF far exceeds that of other clusters. Cluster 4 also exhibits relatively high contributions from RD and SVF, but the intensity is weaker than in Cluster 3, consistent with its transitional nature. In contrast, the dominant low-to-medium value samples (Cluster 1 and Cluster 2) generally show lower and more balanced feature contributions, indicating that the response mechanism for mainstream samples is more attenuated and lacks a single strong driver. Guangzhou exhibits a similar pattern, where RD is established as the primary controlling variable and SVF as the secondary driver, while variables such as PSV, DTT, and BCD play supplementary explanatory roles across different clusters. The spatial scatter plots demonstrate that the clusters possess clear spatial agglomeration, suggesting that the model’s explanatory structure is highly coupled with the heterogeneity of the intra-urban physical space. The cluster mean feature profiles quantify the physical attributes of each cluster (Figure 11).

**Figure 12:**
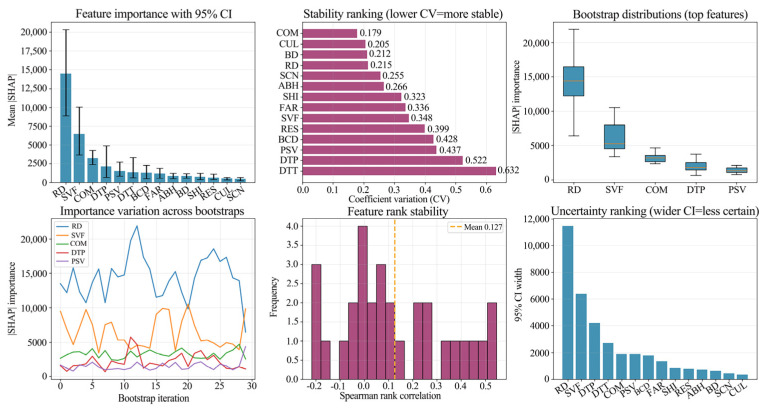
Uncertainty and stability analysis of feature importance in Beijing: 95% CI, coefficient of variation (CV), and rank stability.

**Figure 13:**
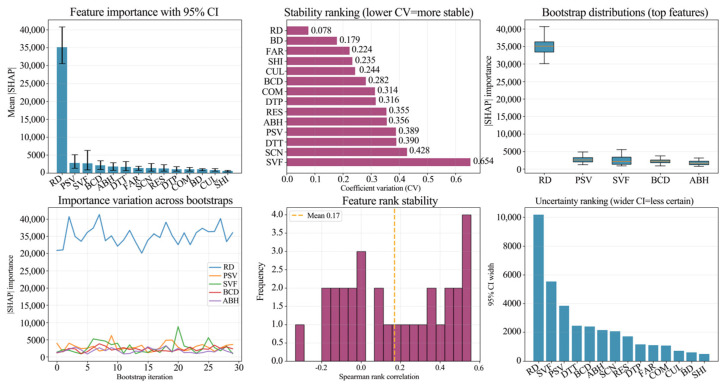
Uncertainty and stability analysis of feature importance in Guangzhou: 95% CI, coefficient of variation (CV), and rank stability.

To rigorously assess the robustness of the SHAP interpretation under sample perturbation, a bootstrap resampling approach was implemented. This involved calculating the 95% Confidence Intervals (CI) for mean SHAP values, the Coefficients of Variation (CV) for feature importance, and Spearman’s rank correlations to evaluate feature rank stability. The results reveal a consistent hierarchical structure across both cities: prominent primary factors underpinned by interchangeable secondary factors. RD emerges as the paramount driver in both metropolises, sustaining the highest SHAP contributions. However, the concentration of influence differs (Figure 12 and Figure 13). Guangzhou displays a traffic corridor dominated regime where RD’s weight far exceeds that of other variables. Beijing, conversely, exhibits a diversified driving mechanism, where explanatory power is shared among a spectrum of urban morphology and functional indicators.

An analysis of uncertainty and stability highlights the following: While top-tier variables maintained their leading positions across resampling, the CI widths and CV rankings uncovered varying degrees of sensitivity. In Guangzhou, RD demonstrates a high importance and low CV profile, suggesting a robust contribution that is resilient to sample noise. In Beijing, transport accessibility and specific morphological/functional variables exhibit higher CVs and wider CIs. This indicates a higher susceptibility of marginal contributions to sample structure, corroborating the stronger spatial non-stationarity inherent in Beijing’s ALAN driving forces.

In terms of rank stability, although rank correlations remained generally stable, crossover and reordering were observed among middle- and lower-ranked variables, whereas core factors like RD remained invariant. Conclusively, this robustness check validates the consistency of the identified primary driver (RD) and key morphological factor (SVF), while attributing the inter-city differences to the composition and stability of the secondary driving architecture.

## 5. Discussion

### 5.1. Key Drivers of Street-Scale ALAN

Based on SDGSAT-1 high-resolution nighttime light data, this study investigates the spatial heterogeneity of ALAN at the street scale in two typical Chinese cities—Beijing and Guangzhou. By integrating the GWRF model with SHAP, we provide an in-depth analysis of the multiple driving factors of ALAN intensity and their spatial variability.

We found that RD is the primary driving factor for ALAN intensity in both cities. At the street scale, road density functions as a fundamental structural proxy for urban spatial organization. Rather than simply reflecting where lights are installed, dense road networks represent areas of high spatial connectivity, mobility intensity, and concentration of human activities. In this sense, RD captures the underlying organization of nighttime urban dynamics, where transportation corridors act as the primary carriers of traffic flows, commercial activities, and social interactions. Therefore, the strong explanatory power of RD indicates that ALAN is not only a product of lighting facilities, but also a spatial manifestation of urban activity patterns.

Furthermore, the dominance of RD reveals distinct urban development logics. In cities such as Guangzhou, where the model shows an overwhelming dependence on RD, nighttime light distribution follows a clear corridor-oriented pattern. This suggests that nighttime economic activities, including retail, entertainment, and logistics, are highly concentrated along major transportation axes. In contrast, Beijing exhibits a more balanced multi-factor structure, implying that its nighttime activities are more spatially diversified and influenced by a broader combination of morphology, function, and accessibility.

### 5.2. Differences in ALAN Formation Mechanisms Between Cities

Beyond the statistical differences revealed by the model, the contrasting ALAN formation mechanisms between Beijing and Guangzhou can also be interpreted in the context of their distinct urban development strategies and socio-economic structures. Beijing has been undergoing a spatial restructuring process aimed at transforming its traditional monocentric structure into a polycentric metropolitan system. Policies such as the relocation of non-capital functions and the coordinated development of the Beijing–Tianjin–Hebei region have promoted the emergence of multiple sub-centers and diversified urban functional zones. As a result, nighttime activities and lighting demand are distributed across various urban contexts, including residential areas, public services, and transportation corridors, leading to a relatively balanced multi-factor driving mechanism for ALAN. In contrast, Guangzhou’s nighttime light pattern reflects its role as a major commercial and transportation hub within the Guangdong–Hong Kong–Macao Greater Bay Area. The city’s spatial structure is strongly shaped by transportation infrastructure and commercial clusters, with dense road networks supporting active nighttime economic activities such as retail and entertainment. Consequently, road density exerts a stronger influence on ALAN intensity, resulting in the traffic-dominated mechanism identified in the model.

### 5.3. Implications for Urban Lighting Planning and Management

Beyond explaining the formation mechanisms of ALAN, the results also provide practical implications for urban lighting planning and management. The SHAP analysis reveals that the contributions of key variables such as road density and sky view factor exhibit clear threshold and nonlinear characteristics, which can support more refined and spatially differentiated lighting strategies.

For example, the SHAP dependence results show that the contribution of RD to ALAN intensity in Beijing follows an inverted U-shaped pattern, with a threshold occurring when RD reaches a certain level. This indicates that in areas with extremely dense road networks, nighttime lighting may already approach saturation. In such cases, simply increasing lighting infrastructure may yield limited benefits. Instead, lighting optimization measures, such as adaptive lighting control, smart street lighting systems, or energy-efficient lamps, may be more effective in improving lighting efficiency while reducing energy consumption.

In addition, the influence of SVF suggests that urban morphology significantly affects the diffusion and visibility of nighttime lighting. Streets with high SVF values, typically characterized by open spaces and wide road corridors, tend to exhibit stronger ALAN intensity. Conversely, narrow streets within dense residential blocks often show lower lighting levels due to building occlusion. These findings imply that lighting planning should consider the spatial form of streets. For instance, in enclosed street canyons, optimizing the placement and height of lighting fixtures may be more effective than simply increasing lamp density.

Furthermore, the clustering results based on SHAP values reveal distinct groups of streets with different driving mechanisms of ALAN intensity. High-intensity clusters are often associated with transportation corridors and open urban spaces, whereas low- and medium-intensity clusters tend to correspond to residential or mixed-function areas. Mapping these cluster patterns provides a useful tool for identifying street segments that may require lighting optimization. For example, areas with relatively low ALAN intensity but high functional activity may require targeted improvements to enhance nighttime safety and accessibility, while high-intensity clusters may prioritize energy-saving strategies and light pollution control.

### 5.4. Limitations and Future Research

Despite the explorations made in this study regarding street-scale ALAN driving mechanisms and the methodological framework, several limitations remain that warrant further improvement in future research.

First, the study covers only two megacities, which limits the universality of the conclusions. Beijing and Guangzhou are both large, economically developed coastal/inland metropolises in China with similar high-intensity development contexts. They cannot fully represent small and medium-sized cities, resource-based cities, mountainous cities, coastal tourist cities, or cities in different climatic and economic zones. Therefore, the conclusions about the dominant role of road density and the differential effects of urban morphology are only applicable to large megacities with similar functional and spatial characteristics, and cannot be directly extended to all city types. Future research should select a more diverse sample of cities with different population sizes, geographical locations, industrial structures, and spatial forms to verify the stability of the analytical framework and the generalizability of driving mechanisms.

Second, the analysis relies on single-time-point nighttime light data, ignoring temporal dynamics. This study uses SDGSAT-1 data from a single period, which cannot capture seasonal variations, weekday–weekend differences, holiday fluctuations, or long-term temporal patterns of urban ALAN. Factors such as temperature, residential activity rhythm, commercial operation hours, and seasonal lighting policies may cause obvious temporal changes in ALAN intensity and spatial distribution. Future studies should construct multi-temporal and long-sequence nighttime light datasets to reveal the spatiotemporal coupling relationship between urban ALAN and human activity patterns.

Third, due to the lack of concurrent in situ luminance measurements, direct validation of SDGSAT-1 radiance was not feasible, and potential atmospheric effects (such as aerosol scattering) remain unquantified, highlighting the need for ground-based observations in future studies.

Fourth, concerning the system of explanatory variables, although this study integrates POI, building, and road network data to construct indicators across the dimensions of morphology, function, and transportation, it focuses primarily on spatial structural characteristics. It does not explicitly incorporate socio-economic variables, such as resident income, electricity load, and industrial structure, nor does it account for institutional factors like lighting standards and energy-saving policies. Future studies could further introduce socio-economic and institutional variables, subject to data privacy and availability, to enhance the explanatory adequacy regarding ALAN heterogeneity.

In conclusion, by innovatively integrating the GWRF model with the SHAP method, this study offers a new perspective for exploring the spatial heterogeneity and formation mechanisms of urban nighttime light. With the continuous enrichment of high-resolution nighttime light data and the further optimization of analytical methods, urban nighttime light research will provide more precise support for urban planning, light environment management, and the monitoring of socio-economic activities. Finally, this study demonstrates that the SHAP method offers robust support for understanding the spatial variation of ALAN. Through SHAP, we were able to quantify the marginal contribution of individual factors to ALAN intensity and reveal interaction effects among them. While road density remains the most critical factor in both Beijing and Guangzhou, the relative importance of other factors, such as SVF, commercial density, and public service density, varies across different regions. These results suggest that future urban ALAN analyses should account for local variations within different areas to avoid simplistic global assumptions.

## 6. Conclusions

This study investigated the spatial drivers of artificial light at night (ALAN) at the street-unit level in Beijing and Guangzhou by integrating high-resolution nighttime light data with built-environment indicators. The results indicate that urban morphology, functional structure, and transportation accessibility jointly influence the spatial distribution of ALAN, while their relative contributions vary across locations due to spatial heterogeneity. By applying the geographically weighted random forest (GWRF) model and explainable machine learning techniques, this study revealed both the predictive relationships and the spatially varying effects of different urban factors on nighttime light intensity.

Beyond the empirical findings, this research contributes to a better understanding of how urban built environments shape nighttime lighting patterns in rapidly urbanizing cities. The results provide useful insights for urban planning and lighting management, highlighting the importance of considering spatial heterogeneity when developing sustainable urban lighting strategies. Moreover, the proposed modeling framework combining spatial machine learning and interpretability analysis offers a practical approach for examining ALAN dynamics in other urban contexts. Future research could further explore temporal variations in nighttime lighting and incorporate additional environmental or socio-economic factors to better understand the long-term impacts of urban lighting systems.

## Figures and Tables

**Table 1:** Spectral wavelength ranges, radiometric gain coefficients, and bias offsets for the three multispectral bands of SDGSAT-1, used for DN-to-radiance conversion.

Band	Wavelength	Gain	Bias
Band_R	424–526 nm	0.0000099253	0.0000099253
Band_G	506–612 nm	0.0000050700	0.0000060840
Band_B	600–894 nm	0.0000135400	0.0000136754

**Table 2:** Indicators and abbreviations related to urban morphology, urban functions, and transportation accessibility.

Dimensionality	Indicators	Abbreviation
Urban morphology	Floor area ratio	FAR
	Average building height	ABH
	Building density	BD
	Building concentration density	BCD
	Sky view factor	SVF
Functional structure	Commercial density	COM
	Public service density	PSV
	Residential density	RES
	Scenery density	SCN
	Cultural density	CUL
	Shannon index	SHI
Transportation accessibility	Road density	RD
	Distance to parking	DTP
	Distance to transport station	DTT

**Table 3:** The formulas and significance of indicators related to urban morphology.

Metrics	Formula and Description	Significance
Floor area ratio	$FAR=\frac{\sum_{i=1}^{n}A_{i}F_{i}}{S}$ $A_{i}$ represents the base area of the building i. $F_{i}$ represents the number floors of building i. S represents the area of the block.	FAR measures the total built area (the sum of the floors of all buildings in a given area) relative to the area of the land (block or plot) on which they are built.
Average building height	$ABH=\sum_{i=0}^{n}H_{i}/n$ $H_{i}$ represents the height of building i. n represents the number of the buildings.	This measure is typically calculated by summing the heights of all buildings in a given area and then dividing by the total number of buildings in that area.
Building density	$BD=\frac{\sum_{i=1}^{n}A_{i}}{S}$ $A_{i}$ represents the base area of the building i. S represents the area of the block.	Building density is a metric used to assess the concentration of buildings within a specific area or block.
Building concentration density	$BCD=\frac{\sum_{i=1}^{n}H_{i}}{S\times H_{max}}$ $H_{i}$ represents the height of building i. S represents the area of the block. $H_{max}$ represents maximum building height	Building concentration density is a metric used to assess the vertical concentration of buildings within a specific area or block.
Sky view factor	$SVF=1-\frac{\sum_{i=1}^{n}sin\varphi_{i}}{n}$ $\varphi_{i}$ represents the impact of the terrain height angle and building orientation on the azimuth angle i, with n denoting the total number of azimuth angles calculated.	The SVF ranges from 0 to 1, with 1 indicating an unobstructed sky (completely open) and 0 indicating a completely obstructed sky (fully covered by buildings).

**Table 4:** Reclassification of POIs.

Reclassified Species	Types of Data
Commercial district	Catering services, corporate enterprises, financial services, shopping services, automobile sales, residential life services.
Culture district	Science and education services, cultural facilities, schools, research institutes.
Residential district	Residential buildings.
Scenery district	Landscape architecture, historic sites, parks, green spaces.
Public service district	Sports and leisure services, healthcare services, administrative and community service centers, public facilities, transport hub services.

## Data Availability

Data will be made available on request.

## References

[1] Kyba C.C., Kuester T., Sánchez de Miguel A., Baugh K., Jechow A., Hölker F., Bennie J., Elvidge C.D., Gaston K.J., Guanter L. (2017). Artificially lit surface of Earth at night increasing in radiance and extent. Sci. Adv..

[2] Li F., Yan Q., Bian Z., Liu B., Wu Z. (2020). A POI and LST adjusted NTL urban index for urban built-up area extraction. Sensors.

[3] Li X., Zhou Y. (2017). Urban mapping using DMSP/OLS stable night-time light: A review. Int. J. Remote Sens..

[4] Liu Y., Yang Y., Jing W., Yao L., Yue X., Zhao X. (2017). A new urban index for expressing inner-city patterns based on MODIS LST and EVI regulated DMSP/OLS NTL. Remote Sens..

[5] Zhao Z., Tang X., Wang C., Cheng G., Ma C., Wang H., Sun B. (2023). Analysis of the spatial and temporal evolution of the GDP in Henan Province based on nighttime light data. Remote Sens..

[6] Wang J., Lu F. (2021). Modeling the electricity consumption by combining land use types and landscape patterns with nighttime light imagery. Energy.

[7] Chakraborty S., Alexander O., Kittner N., Wang Z. (2025). A global stocktake on electricity access and gaps from NASA Black Marble nighttime lights. Earth’s Future.

[8] Liu H., Wang J., Liu H., Chen Y., Liu X., Guo Y., Huang H. (2022). Identification of relative poverty based on 2012–2020 NPP/VIIRS night light data: In the area surrounding Beijing and Tianjin in China. Sustainability.

[9] Huang C., Zhuang Q., Meng X., Guo H., Han J. (2021). An improved nightlight threshold method for revealing the spatiotemporal dynamics and driving forces of urban expansion in China. J. Environ. Manag..

[10] Bara C., Sticher V. (2025). The rural limits of conflict monitoring using nighttime lights. Humanit. Soc. Sci. Commun..

[11] Li Y., Guo W., Li P., Zhao X., Liu J. (2023). Exploring the spatiotemporal dynamics of CO2 emissions through a combination of nighttime light and MODIS NDVI data. Sustainability.

[12] Guo W., Liu J., Zhao X., Hou W., Zhao Y., Li Y., Sun W., Fan D. (2023). Spatiotemporal dynamics of population density in China using nighttime light and geographic weighted regression method. Int. J. Digit. Earth.

[13] Chen Z., Yu B., Yang C., Zhou Y., Qian X., Wang C., Wu B., Wu J. (2020). An extended time-series (2000–2018) of global NPP-VIIRS-like nighttime light data from a cross-sensor calibration. Earth Syst. Sci. Data Discuss..

[14] Guo J., Zhang F., Zhao H., Pan B., Mei L. (2025). Global reconstruction of three decades of fine-grained nighttime light data with analysis of large-scale infrastructure and landmarks. Remote Sens. Environ..

[15] Guo W., Zhao Y., Kyba C.C., Zhao X., Cui X., Liu J., Gao X., Chao M., Wei X., Liu H. (2025). Using a deep learning method to establish 500 m resolution non-saturation nighttime light time series from 1992 to 2022. Int. J. Appl. Earth Obs. Geoinf..

[16] Levin N., Kyba C.C., Zhang Q., de Miguel A.S., Román M.O., Li X., Portnov B.A., Molthan A.L., Jechow A., Miller S.D. (2020). Remote sensing of night lights: A review and an outlook for the future. Remote Sens. Environ..

[17] Guo W., Lu D., Wu Y., Zhang J. (2015). Mapping impervious surface distribution with integration of SNNP VIIRS-DNB and MODIS NDVI data. Remote Sens..

[18] Man D.C., Tsubasa H., Fukui H. (2021). Normalization of VIIRS DNB images for improved estimation of socioeconomic indicators. Int. J. Digit. Earth.

[19] Guo W., Zhang Y., Gao L. (2018). Using VIIRS-DNB and landsat data for impervious surface area mapping in an arid/semiarid region. Remote Sens. Lett..

[20] Li K., Chen Y. (2018). A Genetic Algorithm-based urban cluster automatic threshold method by combining VIIRS DNB, NDVI, and NDBI to monitor urbanization. Remote Sens..

[21] Xie Q., Cai C., Jiang Y., Zhang H., Wu Z., Xu J. (2024). Investigating the performance of SDGSAT-1/GIU and NPP/VIIRS nighttime light data in representing nighttime vitality and its relationship with the built environment: A comparative study in Shanghai, China. Ecol. Indic..

[22] Li C., Chen F., Wang N., Yu B., Wang L. (2023). SDGSAT-1 nighttime light data improve village-scale built-up delineation. Remote Sens. Environ..

[23] Levin N. (2025). Challenges in remote sensing of night lights–a research agenda for the next decade. Remote Sens. Environ..

[24] Chen Z., Luo H., Li M., Lin J., Zhang X., Li S. (2025). Fine-scale poverty estimation by integrating SDGSAT-1 glimmer images and urban functional zoning data. Remote Sens. Environ..

[25] Xu N., Xu Y., Yan Y., Guo Z., Wang B., Zhou X. (2022). Evaluating road lighting quality using high-resolution JL1-3B nighttime light remote sensing data: A case study in nanjing, China. Remote Sens..

[26] Zheng Q., Weng Q., Huang L., Wang K., Deng J., Jiang R., Ye Z., Gan M. (2018). A new source of multi-spectral high spatial resolution night-time light imagery—JL1-3B. Remote Sens. Environ..

[27] Guo B., Hu D., Zheng Q. (2023). Potentiality of SDGSAT-1 glimmer imagery to investigate the spatial variability in nighttime lights. Int. J. Appl. Earth Obs. Geoinf..

[28] Guo H., Dou C., Chen H., Liu J., Fu B., Li X., Zou Z., Liang D. (2023). SDGSAT-1: The world’s first scientific satellite for sustainable development goals. Sci. Bull..

[29] Levin N., Cooper R.M., Kark S. (2024). Quantifying night sky brightness as a stressor for coastal ecosystems in Moreton Bay, Queensland. Remote Sens..

[30] Liu S., Wang C., Wu B., Chen Z., Zhang J., Huang Y., Wu J., Yu B. (2024). Integrating NTL intensity and building volume to improve the built-up areas’ extraction from SDGSAT-1 GLI data. Remote Sens..

[31] Zhang L., Guo H., Liang D., Lv Z., Li Z., Geng Y., Liu X., Lv M., Dou C. (2025). A study on detection of human activity using SDGSAT-1 glimmer imager data over urban agglomerations in China. Remote Sens. Environ..

[32] Wu B., Wang Y., Huang H., Liu S., Yu B. (2024). Potential of SDGSAT-1 nighttime light data in extracting urban main roads. Remote Sens. Environ..

[33] Wang Y., Huang H., Wu B. (2024). Evaluating the Potential of SDGSAT-1 Glimmer Imagery for Urban Road Detection. IEEE J. Sel. Top. Appl. Earth Obs. Remote Sens..

[34] Lv Z., Guo H., Zhang L., Liang D., Gong L., Liu Y. (2025). Advancing urban connectivity measurements for SDG 11. a with SDGSAT-1 nighttime light data in urban agglomerations. Int. J. Appl. Earth Obs. Geoinf..

[35] Alahmadi M., Mansour S., Martin D., Atkinson P.M. (2021). An improved index for urban population distribution mapping based on nighttime lights (DMSP-OLS) data: An experiment in Riyadh Province, Saudi Arabia. Remote Sens..

[36] Xiao Q.-L., Wang Y., Zhou W.-X. (2021). Regional economic convergence in China: A comparative study of nighttime light and GDP. Front. Phys..

[37] Wang F., Zhou K., Wang M., Wang Q. (2020). The impact analysis of land features to JL1-3B nighttime light data at parcel level: Illustrated by the case of Changchun, China. Sensors.

[38] Zheng M., Huang W., Xu G., Li X., Jiao L. (2023). Spatial gradients of urban land density and nighttime light intensity in 30 global megacities. Humanit. Soc. Sci. Commun..

[39] Huang X., Yang J., Li J., Wen D. (2021). Urban functional zone mapping by integrating high spatial resolution nighttime light and daytime multi-view imagery. ISPRS J. Photogramm. Remote Sens..

[40] Liu S., Zhao X., Zhang F., Qiu A., Chen L., Huang J., Chen S., Zhang S. (2022). Spatial downscaling of NPP-VIIRS nighttime light data using multiscale geographically weighted regression and multi-source variables. Remote Sens..

[41] Ye Y., Huang L., Zheng Q., Liang C., Dong B., Deng J., Han X. (2021). A feasible framework to downscale NPP-VIIRS nighttime light imagery using multi-source spatial variables and geographically weighted regression. Int. J. Appl. Earth Obs. Geoinf..

[42] Wu J., Tu Y., Chen Z., Yu B. (2022). Analyzing the spatially heterogeneous relationships between nighttime light intensity and human activities across Chongqing, China. Remote Sens..

[43] Guo B., Bian Y., Zhang D., Su Y., Wang X., Zhang B., Wang Y., Chen Q., Wu Y., Luo P. (2021). Estimating socio-economic parameters via machine learning methods using Luojia1-01 nighttime light remotely sensed images at multiple scales of China in 2018. IEEE Access.

[44] Qin Z., Peng Q., Jin C., Xu J., Xing S., Zhu P., Yang G. (2025). Geographically weighted random forest fusing multi-source environmental covariates for spatial prediction of soil heavy metals. Environ. Pollut..

[45] Luo Y., Yan J., McClure S.C., Li F. (2022). Socioeconomic and environmental factors of poverty in China using geographically weighted random forest regression model. Environ. Sci. Pollut. Res..

[46] Kurniati B., Dewi Y.S., Hadi A.F. (2023). Handling multicollinearity on social spatial data using geographically weighted random Forest. SAR J.-Sci. Res..

[47] Su Z., Lin L., Xu Z., Chen Y., Yang L., Hu H., Lin Z., Wei S., Luo S. (2023). Modeling the effects of drivers on PM2. 5 in the Yangtze River Delta with geographically weighted Random Forest. Remote Sens..

[48] Li Z. (2022). Extracting spatial effects from machine learning model using local interpretation method: An example of SHAP and XGBoost. Comput. Environ. Urban Syst..

[49] Zhang J., Ma X., Zhang J., Sun D., Zhou X., Mi C., Wen H. (2023). Insights into geospatial heterogeneity of landslide susceptibility based on the SHAP-XGBoost model. J. Environ. Manag..

[50] Wang X., Zhang Y., Li C., Yin C., Shao C. (2025). Investigating nonlinear and spatially heterogeneous impacts of the built environment on urban vitality. Sustain. Cities Soc..

[51] Li Z., Chen B., Huang Y., Wang H., Wang Y., Yuan Y., Li X., Chen J.M., Xu B., Gong P. (2025). Enhanced mapping of essential urban land use categories in China (EULUC-China 2.0): Integrating multimodal deep learning with multisource geospatial data. Sci. Bull..

[52] (2017). Classification of Land Use Status.

[53] Grundland M., Dodgson N.A. (2007). Decolorize: Fast, contrast enhancing, color to grayscale conversion. Pattern Recognit..

[54] Zou B., Xu S., Liu N., Li S., Liu X., Guo Y., Zhan F.B. (2023). PM_2.5_ exposure and associated premature mortality to 2100 in China under climate and socioeconomic change scenarios. Earth’s Future.

[55] Georganos S., Kalogirou S. (2022). A forest of forests: A spatially weighted and computationally efficient formulation of geographical random forests. ISPRS Int. J. Geo-Inf..

[56] Zhou Y., Wei G., Wang Y., Wang B., Quan Y., Wu Z., Liu J., Bian S., Li M., Fan W. (2025). Estimating Regional Forest Carbon Density Using Remote Sensing and Geographically Weighted Random Forest Models: A Case Study of Mid-to High-Latitude Forests in China. Forests.

